# Preclinical Models of Traumatic Brain Injury: Emerging Role of Glutamate in the Pathophysiology of Depression

**DOI:** 10.3389/fphar.2018.00579

**Published:** 2018-06-01

**Authors:** Darik A. O’Neil, Melissa A. Nicholas, Naima Lajud, Anthony E. Kline, Corina O. Bondi

**Affiliations:** ^1^Physical Medicine & Rehabilitation, University of Pittsburgh, Pittsburgh, PA, United States; ^2^Safar Center for Resuscitation Research, University of Pittsburgh, Pittsburgh, PA, United States; ^3^División de Neurociencias, Centro de Investigación Biomédica de Michoacán – Instituto Mexicano del Seguro Social, Morelia, Mexico; ^4^Center for Neuroscience, University of Pittsburgh, Pittsburgh, PA, United States; ^5^Center for the Neural Basis of Cognition, University of Pittsburgh, Pittsburgh, PA, United States; ^6^Critical Care Medicine, University of Pittsburgh, Pittsburgh, PA, United States; ^7^Department of Psychology, University of Pittsburgh, Pittsburgh, PA, United States; ^8^Department of Neurobiology, University of Pittsburgh, Pittsburgh, PA, United States

**Keywords:** traumatic brain injury (TBI), depression, glutamatergic neurotransmission, animal models, pharmacotherapies

## Abstract

More than 10 million people worldwide incur a traumatic brain injury (TBI) each year, with two million cases occurring in the United States. TBI survivors exhibit long-lasting cognitive and affective sequelae that are associated with reduced quality of life and work productivity, as well as mental and emotional disturbances. While TBI-related disabilities often manifest physically and conspicuously, TBI has been linked with a “silent epidemic” of psychological disorders, including major depressive disorder (MDD). The prevalence of MDD post-insult is approximately 50% within the 1st year. Furthermore, given they are often under-reported when mild, TBIs could be a significant overall cause of MDD in the United States. The emergence of MDD post-TBI may be rooted in widespread disturbances in the modulatory role of glutamate, such that glutamatergic signaling becomes excessive and deleterious to neuronal integrity, as reported in both clinical and preclinical studies. Following this acute glutamatergic storm, regulators of glutamatergic function undergo various manipulations, which include, but are not limited to, alterations in glutamatergic subunit composition, release, and reuptake. This review will characterize the glutamatergic functional and signaling changes that emerge and persist following experimental TBI, utilizing evidence from clinical, molecular, and rodent behavioral investigations. Special care will be taken to speculate on how these manipulations may correlate with the development of MDD following injury in the clinic, as well as pharmacotherapies to date. Indisputably, TBI is a significant healthcare issue that warrants discovery and subsequent refinement of therapeutic strategies to improve neurobehavioral recovery and mental health.

## Introduction

Traumatic brain injuries (TBI) have recently soared into notoriety (Goldstein, 1990; Selassie et al., 2008), with over one million TBI-related emergency room visits in the United States alone yearly (Faul et al., 2010). Over 40% of Americans have TBI-related disabilities following hospitalization (Selassie et al., 2008; Summers et al., 2009) and there is a scarcity of viable treatment options despite their sequalae affecting a diverse collection of quality of life parameters. One of the most common comorbidities in TBI patients has been major depressive disorder (MDD) with a varied and multifactorial etiology which to date, remains poorly defined (Dean and Keshavan, 2017). The prevalence of MDD post-insult has been reported as being nearly 50% within the 1st year (Bombardier et al., 2010). While the emergence of MDD post-insult is almost certainly intertwined with a variety of psychogenic and pathophysiological factors, such as stress exposure (Hammen, 2005), leading to poor global and psychosocial outcome, as well as cognitive compromise (Rapoport, 2012), distinct TBI-related dysfunction in the brain may drive the manifestation of depression beyond the expected rate of emergence. Moreover, the pathophysiological landscape of both MDD and TBI is marred by widespread disturbances in glutamatergic functioning (Sanacora et al., 2012; Guerriero et al., 2015). Given its activity as the primary excitatory brain neurotransmitter (Zhou and Danbolt, 2014), glutamate may play a significant role in orchestrating the vulnerability of the post-TBI brain. This review seeks to construct a roadmap of glutamatergic dysfunction following TBI and to elucidate the implications of these alterations in the context of therapeutics and neurobehavioral recovery.

## Preclinical Models of TBI

Preclinical rodent models have proven instrumental in elucidating a systemically progressive pathophysiology, where abnormalities of membrane polarization and ionic constituency evolve into metabolic crises, altered neurotransmitter function, and cellular death (Bondi et al., 2015). In humans, TBI is a remarkably heterogenous disease, recently necessitating a multisite research consortium initiative by the U.S. Army entitled “Operation Brain Trauma Therapy” to conduct an extensive analysis of pharmacological interventions across multiple preclinical models (Kochanek et al., 2016). Experimental models of TBI have been developed to study injury biomechanics, discover pathological mechanisms, and develop therapies with the goal of reducing TBI-induced human suffering (Bondi et al., 2015). The controlled cortical impact (CCI) is a focal injury model that has seen widespread implementation in preclinical research. Typically, researchers perform a craniectomy and subsequently use a piston to apply a controlled impact to the dura matter, causing damage to the underlying tissue (Dixon et al., 1991). Manipulations to the depth, speed, location, and number of impacts have allowed researchers to perform a wide variety of etiological and severity-based assessments (Budinich et al., 2012; Washington et al., 2012; Bondi et al., 2014). The fluid percussion injury model (FPI) is another highly prolific model, involving diffuse or mixed (diffuse and focal) injuries. The injury is induced utilizing a fluid pressure chamber, which is struck by a pendulum. A pulse of pressure strikes the exposed dura mater, at the midline sagittal suture (i.e., rendering concussive injuries) (Dixon et al., 1987) or laterally over parietal cortex (i.e., rendering hippocampal cell death and cortical contusions) (McIntosh et al., 1989). Much like the CCI, injury severity and the number of fluid pulses can be adjusted. In weight-drop models of TBI, the skull is impacted with a free-falling weight on a guided path. Varying models exist, such as a focal impact applied to the intact skull (Hall et al., 1988), the Marmarou impact acceleration model (Marmarou et al., 1994), or the head rotation model (Ross et al., 1994), and the injury severity can be modified by manipulating the initial height and the mass of the weight. Blast TBI injury models, mainly diffuse in nature, are clinically and biomechanically relevant to military TBIs, by exposing animals to explosive or pressurized gas-driven shock tubes to generate pressure waves similar to those produced by explosives (Svetlov et al., 2010).

## Glutamatergic Alterations in TBI

Glutamate was first described as a neurotransmitter in 1954, much later than the other classical neurotransmitters, in part due to its relative abundance and complex web of non-synaptic interactions (Hayashi, 1954). At points of synaptic contact, concentrations of glutamate are only in the nanomolar (nM) magnitude (Moussawi et al., 2011). Meanwhile, intracellular concentrations are approximately 10 mM, while cerebrospinal fluid levels are estimated in the range of 10 μm (Moussawi et al., 2011). In the decades since, glutamate has been revealed as the primary excitatory neurotransmitter in the CNS. Vesicular transporters (VGLUT 1-3) transport intracellular glutamate into synaptic vesicles, which are released in a calcium-dependent manner utilizing the SNARE complex mechanism (Hamberger et al., 1978; Liguz-Lecznar and Skangiel-Kramska, 2007; Popoli et al., 2011). Glutamate can be detected by three types of ligand-gated ionotropic receptors: α-amino-3-hydroxy-5-methyl-4-isoxazolepropionic acid (AMPA), with GluA1-2 combinations being the most prominent in the rodent, at least in the hippocampus (Lu et al., 2009; Henley and Wilkinson, 2013), N-methyl-D-aspartate (NMDA), each with their own variants: GluN1, GluN2, and GluN3 (Cull-Candy and Leszkiewicz, 2004; Iacobucci and Popescu, 2017), and kainite (KA), albeit the latter is comparatively understudied and poorly understood. Electrophysiological studies have established that NMDA receptors do not necessarily impact the peak magnitude of the postsynaptic membrane potential. Instead, the fast AMPAR-mediated current establishes peak magnitude before quickly subsiding, whereas the slower NMDA-mediated current manipulates the temporal decay of the total potential, and thus manipulates the total amount of ionic flux (Iacobucci and Popescu, 2017). Glutamate may also activate three different groups of metabotropic receptors (mGluRs I-III). Group I receptors initiate calcium mobilization and activation of protein kinase C, while group II and III mGluRs are, in contrast, classically coupled to the inhibition of adenylyl cyclase and directly regulate ion channels and other downstream signaling partners (Niswender and Conn, 2010), thus primarily fulfilling autoreceptor roles in the brain. Glutamate reuptake involves five types of excitatory amino acid transporters (EAATs) and the glutamate-cysteine exchanger. Intriguingly, the bulk of glutamate reuptake is accomplished by astrocytes, particularly via EAAT2, where they are converted to glutamine and transported back into neurons or fed into the tricarboxylic acid (TCA) cycle to serve as metabolic fuel (Fontana, 2015; Rose et al., 2016).

In recent years, glutamatergic neurotransmission has emerged as a pivotal player in the pathophysiology of TBI, its dysfunction resonating throughout the acute, subacute, and chronic phases of injury (Dorsett et al., 2017). Moreover, preclinical and clinical research suggests that TBI and MDD share common mechanisms in the dysregulation of glutamate homeostasis (Piao et al., 2017). Following the discovery of potassium disturbances immediately following TBI (Takahashi et al., 1981), the connection between potassium abnormalities, excessive release of excitatory amino acids, and tissue damage was materialized (Hayes et al., 1988; Faden et al., 1989). When sufficient force is rapidly applied to the brain, neurons will undergo concomitant biomechanical injury, generating significant ionic flux and inappropriate depolarization (Povlishock, 1992; Wolf et al., 2001). This initial insult generates an influx of calcium, then glutamate is indiscriminately released at synapses and neuronal activity rapidly proliferates (Kochanek et al., 2015). Provided sufficient insult, what begins as transient depolarization can lead to the activation of apoptotic and non-apoptotic cellular death cascades, followed by significant excitotoxic damage and even cell death (Szydlowska and Tymianski, 2010; Kochanek et al., 2015; Dorsett et al., 2017). With the establishment of excitotoxicity in the pathophysiology of TBI, researchers expanded on initial investigations of astrocytes (Bakay et al., 1977; Kimelberg et al., 1989, 1995). Maxwell and colleagues proposed that astrocytic swelling works to limit glutamate diffusion from the synaptic space—suggesting potential compensation (Maxwell et al., 1994). A short time later, it was revealed that expression of EAAT1 and EAAT2 were reduced at multiple time points following CCI (6***–***72 h post-insult), indicating an attenuation of astrocyte-mediated glutamate uptake (Rao et al., 1998; Kim et al., 2011). In a blast-injury model, altered glutamate uptake was also reported, as the number of EAAT2-containing astrocytes was reduced at 2 h post-insult (Miller et al., 2017), although the overall expression of EAAT2 was unchanged. While it was proposed that the post-traumatic reduction in cellular EAAT1 and EAAT2 expression is predominantly due to degeneration of astrocytes and to downregulation of surviving astrocytes (van Landeghem et al., 2006), recent research has further complicated our understanding of the astrocytic reaction to traumatic insult, with one group noting increases of 20-25% in EAAT4 expression following lateral FPI (3***–***7 days post-insult) (Yi et al., 2007). Nevertheless, more research is needed to clearly elucidate whether specific astrocyte-induced alterations of the glutamatergic system are compensatory or dysfunctional.

Investigations into glutamatergic dysfunction post-injury have also revealed notable changes in receptor expression. Patel and colleagues revealed that GluN2B-subunit containing receptors are particularly vulnerable to mechanical stress, given that neurons containing larger ratios of subunits were more susceptible to injury-induced loss of functional connectivity (Patel et al., 2014). Moreover, broad decreases in GluNR subunit expression across multiple brain regions following lateral FPI (Osteen et al., 2004). It was noted that the ipsilateral occipital and parietal cortices had decreased ratios of GluN2A:GluN2B subunit expression at 1 and 14 days post-insult, and additionally, the bulk of calcium flux was determined via calcium autoradiography to be GluN2B-mediated. Notably, it has been suggested that GluN2A-predominant receptors are more responsible for synaptic reception, while GluN2B-predominant receptors are more responsible for extrasynaptic reception (Kohr, 2006). Hence, it is possible that TBI induces an increase in extrasynaptic signaling. A hippocampal-specific study found acute reductions in GluNRs following CCI (Kumar et al., 2002). Nevertheless, these changes were transient, and expression returned to sham levels within 24 h. Chronically, it appears GluN2B subunits may rebound, and even increase relative to sham levels (Kharlamov et al., 2011). Though not as extensively, AMPA receptor changes have been investigated following injury. Following the flurry of glutamatergic activity that accompanies the insult, GluR1 subunit phosphorylation is significantly upregulated at 4 h, but not 1 or 24 h (Atkins et al., 2006). Furthermore, GluR2-endocytosis appears to occur (Bell et al., 2007, 2009). Overall, it appears that GluR1 subunits decrease, while the expression of GluR2 subunits is not well characterized (Guerriero et al., 2015). The authors of this review suggest that more dedicated research is necessary to explore AMPA receptors following TBI, given that they are coupled to NMDA receptors in plastic responses.

## Mdd and TBI: a Glutamatergic Hypothesis

**FIGURE 1:**
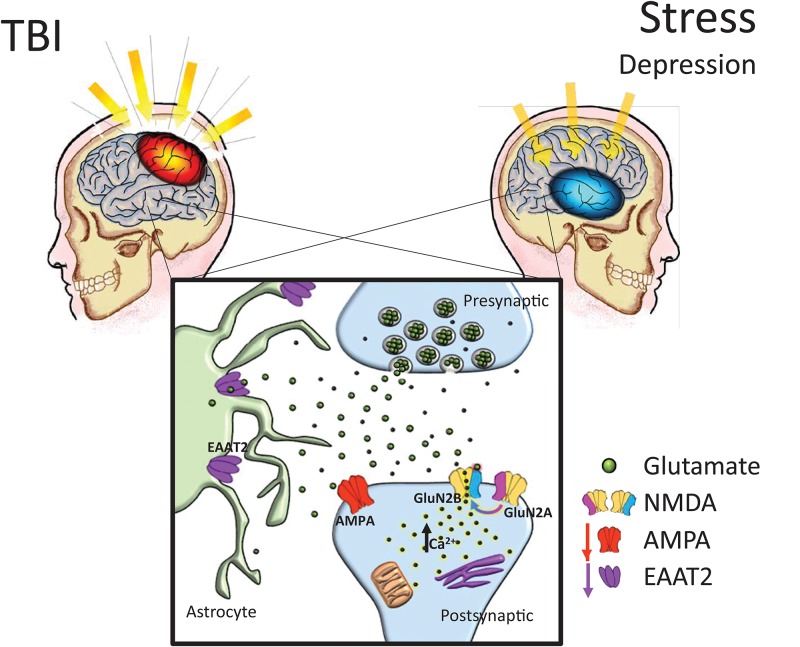
Chronic glutamatergic dysfunction encountered in both TBI and stress-induced depression is notably similar. TBI and stress exposure often are responsible for reductions in a variety of brain glutamatergic markers including EAAT2 and AMPA expression, which in combination with a shift toward GluN2B subunit dominant NMDA receptor composition (i.e., reduced GluN2A:GluN2B ratios), it could lead to increased neuronal vulnerability. AMPA: α-amino-3-hydroxy-5-methyl-4-isoxazolepropionic acid receptor, NMDA: *N*-methyl-D-aspartate receptors, EAAT: excitatory amino acid transporters.

Historically, etiological explanations of depression have specifically involved widespread disturbances of monoamine function (Krishnan and Nestler, 2008). However, current research has suggested that some variations of clinically defined depression may be rooted in dysfunctions of the glutamatergic system (Matthews et al., 2012). The conventional treatment plan for moderate depression has utilized pharmacotherapies which target serotonin functioning, complemented by psychotherapy and psychosocial intervention (Davidson, 2010). While a comprehensive mechanism for the involvement of glutamate in depression has not been universally accepted, there is reasonable evidence suggesting glutamatergic alterations can be observed in many cases of MDD (Sanacora et al., 2012), many of which resembling alterations also reported following TBI (see **Figure 1**). Thompson and colleagues noted reductions in markers of AMPA activity in a plethora of regions after chronic stress, including the nucleus accumbens, prefrontal cortex, and the hippocampus (Thompson et al., 2015). Additionally, shifts from GluN2A to GluN2B subunit expression in the hippocampus were reported in prenatal stress (Sun et al., 2013) and chronic mild stress (Calabrese et al., 2012) models. Intriguingly, in human post-mortem depressed patients, research has revealed that in the amygdala, which is known to become hyperactive in stressful contexts, GluN2A and PSD-95 are increased without concomitant GluN1 increase, suggesting a shift from GluN2B to GluN2A signaling (Karolewicz et al., 2009). Reger and colleagues have shown NMDA receptor upregulation in the rat amygdala following a mild, lateral FPI injury, in stark contrast to the reductions in receptor expression seen in the vast majority of preclinical TBI research (Reger et al., 2012). The characteristic GluN2A to GluN2B subunit shift was again not observed in the amygdala. Furthermore, glutamate reuptake is impaired under chronic stress (de Vasconcellos-Bittencourt et al., 2011), albeit subsequent glutamate release is significantly facilitated in response to acute stress challenges (Popoli et al., 2011).

In rodent models of depression, EAATs are also downregulated in animals displaying depressive behaviors compared to controls (Gomez-Galan et al., 2013), while pharmacologic blockade of glutamate transporters on astrocytes induces anhedonic behaviors in rodents (Bechtholt-Gompf et al., 2010). Recently, Piao et al. (2017) determined that the serum protease, thrombin, may act in CNS via a TBI-compromised blood-brain barrier, on protease-activated receptors in astrocytes, thus causing a decrease in the astrocyte glutamate transporters in the hippocampus, an increase in extracellular glutamate in response to stress, and an increase in depressive-like behavior (e.g., tail-suspension and forced swim tests, sucrose preference test) following TBI. It could thus be speculated that glutamatergic disturbances following TBI underlie vulnerability to depression, where patients’ functional neuroarchitecture and behavioral characteristics mimic the phenotype observed in chronic stress-related iterations of MDD (**Figure 1**).

## Glutamate Receptors: a Gateway to Therapeutics

With the goal of therapeutics to restore cellular changes resulting in the aftermath of TBI (see **Figure 1**), as well as improve functional outcome, studies have typically coalesced molecular investigations with the context of behavioral recovery, providing optimistic outlooks for a variety of glutamatergic compounds. The NMDA-receptor negative allosteric modulator phencyclidine (PCP) was used to investigate long-term gross motor functioning recovery in a FPI model (Hayes et al., 1988). The group reported that PCP pretreatment alleviated TBI-induced deficits in beam balance, beam traversal speed, ambulatory activity, and performance on an incline plane. More recently, the non-competitive NMDA receptor antagonist, gacyclidine, has been examined in a bilateral contusion of the medial frontal cortex, and it similarly improved cognitive performance in the Morris water maze (Smith et al., 2000), while enhancing surviving cell numbers in thalamus, and increasing the size and number of microglia and astrocytes in the striatum. Another non-competitive NMDA receptor antagonist, dizocilpine maleate (MK-801), administered immediately following FPI in immature rats, decreased hippocampal cell loss, diminished memory-related impairments on the novel object recognition test, and decreased anxiety-like responses in the elevated plus maze test 2 months after surgery (Sönmez et al., 2015). Potentially even more promising results came from a different drug from the same class, memantine, which prevented hippocampal neuronal loss when given to rats immediately after CCI injury (Rao et al., 2001), as well as attenuated FPI-induced motor deficits, infarction volume, neuronal loss, and nitrosative stress, while also normalizing the GluN2A/GluN2B ratio by reducing GluN2B expression in the perilesion cortex (Wang et al., 2018). In order to obtain a more clinically translatable TBI treatment approach, other studies combined memantine with neuroprotective compounds, such as the free radical scavenger melatonin, determining that the memantine/melatonin combination in mice decreased TBI-induced DNA fragmentation, p38 phosphorylation and inducible nitric oxide synthase activity compared to each therapy alone (Kelestemur et al., 2016). An interesting study using acute administration of memantine and 17β-estradiol (E2) in rats subjected to FPI reported a reduction in degenerating neurons in hippocampus and cortex, albeit behavioral recovery was not robust enough in order to preferentially endorse this combination therapy (Day et al., 2017). A recent report examined effects of memantine (2× daily for 7 days) in patients with moderate TBI and concluded that they benefited from this treatment as determined by significantly reduced serum levels of neuron-specific enolase, a marker of neuronal damage, as well as improvement in their Glasgow Coma Scale scores compared to the control group (Mokhtari et al., 2018). Although animal findings overall supported the potential of NMDA antagonists as neuroprotective agents after TBI, many clinical studies have unfortunately been mostly inconclusive or terminated prematurely, such as those using selfotel, aptiganel, dexanabinol or EAA 494 (Muir, 2006).

Other potential therapeutic treatments for TBI are centered upon restoring the intracellular concentration of ions that are vital to the glutamatergic signaling pathway. For example, magnesium sulfate effects have been explored in rat models of impact acceleration and FPI. Acute administration of magnesium sulfate has been shown to reduce progressive tissue loss in the hippocampus (Browne et al., 2004), improve depression- and anxiety-like behavior in rats following TBI, such as spontaneous activity test (Fromm et al., 2004), and improve motor and cognitive outcomes using the rotarod and Barnes maze tests, respectively (Turner et al., 2004). In a model of closed-head injury, neurological recovery was enhanced by blood glutamate scavenging following administration of either combination of oxaloacetate/glutamate-oxaloacetate transaminase (Zlotnik et al., 2007) or pyruvate/glutamate-pyruvate transaminase (Zlotnik et al., 2008).

Following FPI, continuous i.v. infusion (72 h) of talampanel, a non-competitive AMPA antagonist, improved pyramidal cell counts in the hippocampal CA1 region (Belayev et al., 2001). Perampanel (5 mg/kg given at 5 min post-injury) also ameliorated CCI-induced brain edema, contusion volumes, and gross motor dysfunction via the beam-balance test, while also suppressing the expression of pro-inflammatory cytokines (TNF-α and IL-1β) and enhancing the levels of anti-inflammatory cytokines (IL-10 and TGF-β1) (Chen et al., 2017), therefore suggesting the importance of AMPA receptors in TBI damage involving secondary injury and inflammation processes. The β-lactam antibiotic ceftriaxone is another promising drug which, when given daily following lateral FPI, had the ability to upregulate glutamate transport, as well as to reestablish EAAT2 expression, reduce seizure activity, and attenuate reactive astrogliosis quantified by glial fibrillary acid protein-expression (Goodrich et al., 2013). Furthermore, ceftriaxone appears to reduce TBI-induced edema formation and improves spatial memory performance in the Morris water maze (Wei et al., 2012; Cui et al., 2014). A few other studies employed the mGluR antagonist, α-methyl-4-carboxyphenylglyicine (MCPG), and reported motor function and spatial memory improvement post-FPI when administered into the rat left ventricle 5 min prior to insult (Gong et al., 1995) or reduced the hybridization signals for immediate early genes c-fos and c-jun mRNA after *in vitro* traumatic injury in glial cells (Katano et al., 2001). Although a variety of compounds appear promising in the laboratory, the translation of preclinical treatments to clinical interventions is still work in progress.

In parallel, despite the growing volume of work investigating glutamatergic disturbances following TBI, there are methodological issues that complicate the translatability and replicability of preclinical rodent studies. Primarily, anesthetics such as ketamine and isoflurane are potent NMDA antagonists, and may operate in a similar manner to pretreatment in the early NMDA antagonist studies, potentially subverting the proper characterization of glutamatergic disturbances (Petrenko et al., 2014). For instance, it has been noted that the presence of NMDA antagonists during insult can attenuate spreading depolarizations (Hertle et al., 2012). Recent studies also suggest glutamatergic drugs, most notably ketamine, show incredible promise in the treatment of depression in both humans and rats (Burger et al., 2016; Jiang et al., 2017). While drugs such as ketamine have been characterized in the context of the intensive care unit, studies assessing their feasibility and impact in the chronic phases of TBI have been fairly scarce (Chang et al., 2013; Zeiler et al., 2014). We thus suggest that when characterizing glutamatergic dysfunction in TBI, anesthetics utilized should ideally not affect the glutamatergic and GABAergic systems.

## Conclusion

The pathophysiology of TBI in rodents is initially characterized by ionic dysfunction and a “storm” of glutamatergic activity. As a result, intracellular calcium levels soar, and through a variety of pathways, cellular death and dysfunction becomes imminent. Astrocytes, the neuronal support system, experience manipulations in their functional activity, best characterized by their inability to clear glutamate from the synaptic space. Subsequently, many brain regions display a shift from synaptic to extrasynaptic ionotropic glutamatergic signaling, as well as broad receptor downregulation. Chronic-stress induced depression is eerily similar to this environment, where glutamatergic clearance is impaired and similar modulations of NMDA and AMPA expression occur. This similarity may underlie a precipitating factor where TBI patients are more vulnerable to developing depression. Nevertheless, more research needs to be performed in both the preclinical and clinical arenas. While researchers have made great progress in illuminating the relationships between TBI, glutamatergic dysfunction, and MDD, we have only scratched the surface of these complex phenomena.

## Author Contributions

All authors participated in writing the manuscript. NL produced the figure. All authors participated in final editing and made intellectual contributions to this work.

## Conflict of Interest Statement

The authors declare that the research was conducted in the absence of any commercial or financial relationships that could be construed as a potential conflict of interest.
